# Supplementary material for: Engineering Synthetic Microbial Communities through a Selective Biofilm Cultivation Device for the Production of Fermented Beverages
Source: Microorganisms. 2019 Jul 20;7(7):206. doi: 10.3390/microorganisms7070206 (PMC6680646; doi:10.3390/microorganisms7070206)
Supplement: Supplementary file 1 [file microorganisms-07-00206-s001.pdf]

Supplementary Materials:

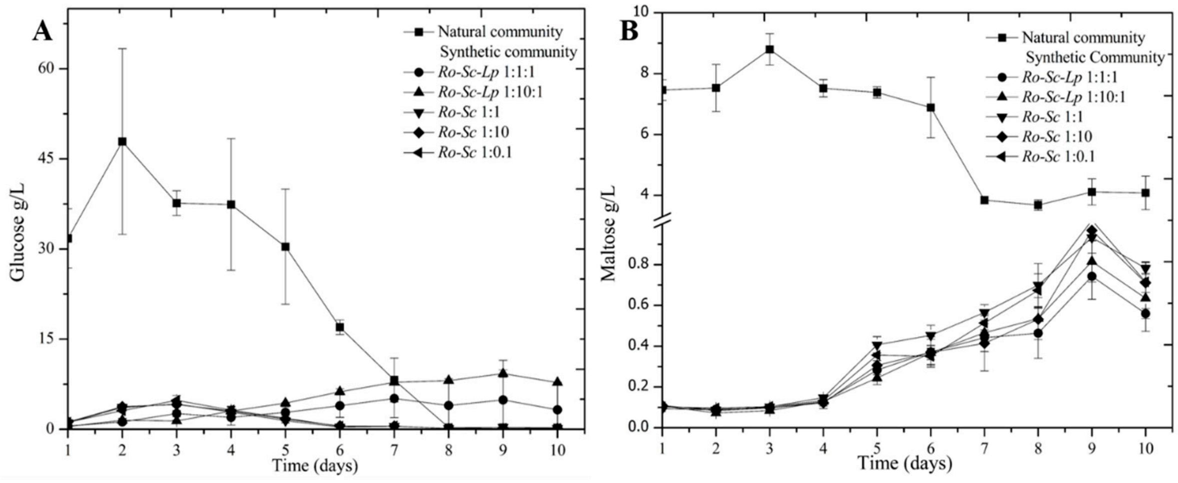

Figure S1. Kinetics of glucose (A) and maltose (B) production during fermentation by natural and synthetic communities. Synthetic communities have been prepared with different ratio between microbial species. In all cases *R. oryzae* (*Ro*) has been inoculated at an initial concentration of 10^6 spores/ml. For the cultures involving either *S. cerevisiae* (*Sc*) or *L. plantarum* (*Lp*), their initial concentration is indicated by 0.1, 1 or 10 corresponding to 10^5, 10^6 and 10^7 cells/mL respectively
